# Enhanced In Vitro Efficacy of Verbascoside in Suppressing Hepatic Stellate Cell Activation via ROS Scavenging with Reverse Microemulsion

**DOI:** 10.3390/antiox13080907

**Published:** 2024-07-27

**Authors:** Xiao Xiao, Feiyu Yang, Yuling Huang, Shaohui Liu, Zhenhua Hu, Shanggao Liao, Yuanyuan Li

**Affiliations:** 1 School of Pharmacy, Guizhou Medical University, Guiyang 550025, China19969984230@163.com (S.L.); 2 Zhongshan Institute for Drug Discovery, Shanghai Institute of Materia Medica, Chinese Academy of Sciences, Zhongshan 528400, China; huzhh@nfu.edu.cn; 3 University Engineering Research Center for the Prevention and Treatment of Chronic Diseases by Authentic Medicinal Materials in Guizhou Province & School of Pharmacy, Guiyang 550025, China; 4 School of Pharmaceutical Sciences, Southern Medical University, Guangzhou 510515, China; 5 Department of Health and Nursing, Nanfang College of Sun Yat-sen University, Guangzhou 510970, China; 6 Shanghai Institute of Materia Medica, Chinese Academy of Sciences, Shanghai 201203, China; 7 University of Chinese Academy of Sciences, Beijing 100049, China

**Keywords:** liver fibrosis, verbascoside, hepatic stellate cells, reverse microemulsion, antioxidants

## Abstract

Numerous approaches targeting hepatic stellate cells (HSCs) have emerged as pivotal therapeutic strategies to mitigate liver fibrosis and are currently undergoing clinical trials. The investigation of herbal drugs or isolated natural active compounds is particularly valuable, due to their multifaceted functions and low risk of side effects. Recent studies have hinted at the potential efficacy of verbascoside (VB) in ameliorating renal and lung fibrosis, yet its impact on hepatic fibrosis remains to be elucidated. This study aims to evaluate the potential effects of VB on liver fibrosis by assessing its ability to inhibit HSC activation. VB demonstrated significant efficacy in suppressing the expression of fibrogenic genes in activated LX-2 cells. Additionally, VB inhibited the migration and proliferation of these activated HSCs by scavenging reactive oxygen species (ROS) and downregulating the AMPK pathway. Furthermore, a biosafe reverse microemulsion loaded with VB (VB-ME) was developed to improve VB’s instability and low bioavailability. The optimal formulation of VB-ME was meticulously characterized, revealing substantial enhancements in cellular uptake, ROS-scavenging capacity, and the suppression of HSC activation.

## 1. Introduction

Hepatic fibrosis, a condition that can progress to cirrhosis, liver failure, and an elevated risk of hepatocellular carcinoma (HCC), poses a growing concern for human health [1,2]. It arises as a fibrous scar due to various chronic liver diseases, including hepatitis C virus infection and non-alcoholic hepatic conditions [3]. At the forefront of liver fibrosis development and progression are hepatic stellate cells (HSCs), the primary contributors to fibrous scar formation [4]. HSC activation induces liver fibrosis by promoting high proliferation, inhibiting apoptosis, accumulating extracellular matrix (ECM), and producing pro-inflammatory cytokines [5]. Targeting HSCs has thus emerged as a pivotal therapeutic strategy to mitigate liver fibrosis [6,7]. While numerous approaches targeting HSCs are undergoing clinical trials, the investigation of herbal drugs or isolated natural active products is particularly valuable due to their multifaceted functions and low risk of side effects [8,9,10].

Verbascoside (VB), also known as Acteoside, was initially isolated from mullein and is also abundantly present in various medicinal herbs and plants [11]. It is a caffeoyl phenylethanoid glycoside that exhibits diverse biological activities, including antioxidant, anti-inflammatory, antimicrobial, and neuroprotective properties [12,13]. While recent studies have suggested its potential efficacy in ameliorating renal and lung fibrosis [14,15,16], its impact on anti-hepatic fibrosis remains to be elucidated. This study aims to evaluate the antifibrotic properties of VB in the liver by examining its inhibitory effects on HSC activation and exploring the corresponding regulatory mechanisms.

Additionally, VB faces challenges such as instability at pH 7.4 [17] and low permeability across cell membranes, resulting in low bioavailability [18,19]. Reverse microemulsion, as a thermodynamically stable system with a straightforward fabrication process, emerges as an ideal carrier for the enhancement of cell permeability of hydrophilic molecules [20,21]. Here, we developed a biosafe reverse microemulsion formulation aimed at amplifying VB’s biological effects by enhancing cellular uptake and improving its stability.

## 2. Materials and Methods

### 2.1. Materials

Isopropyl myristate (IPM) was purchased from Aladdin (Shanghai, China), and phosphatidylcholine (soy) (SoyPC) was obtained from Sigma (Shanghai, China). All chemicals used in this article are of analytical grade. The VB compound originated from the research group of Liao Shanggao at Guizhou Medical University. The Nrf2 antibody was from Huabio (Hangzhou, China, HA721432).

### 2.2. Cell Culture and Treatment

The human hepatic stellate cell line LX-2 was procured from Sigma (St. Louis, MO, USA, SCC064). Cells were cultured in DMEM (Gibco, Waltham, MA, USA) supplemented with 10% fetal bovine serum (FBS, Sigma, St. Louis, MO, USA, F8318-500Ml) and 1% penicillin/streptomycin (Gibco, Waltham, MA, USA, 15140-122) at 37 °C with 5% CO_2_. For the experiments, the cells were starved in 1% FBS medium for 12 h, then pretreated with 50 µM VB or VB-ME for 6 h, and subsequently treated with 5 ng/mL TGFβ1 (Peprotech, Rocky Hill, NJ, USA, 100-21) for 24 h [22]. To inhibit Nrf2, ML385 (MedChemexpress, Monmouth Junction, NJ, USA, HY-100523) was added either alone or in combination with VB for 6 h before TGFβ1 treatment [23]. Following the TGFβ1 treatment for 24 h, the cell samples were collected to measure mRNA levels or stained for immunofluorescence.

The human colon cancer cell line Caco-2 was cultured in high-glucose DMEM medium containing 10% FBS and 1% penicillin/streptomycin. To investigate whether VB-ME promoted the absorption of Caco-2 cells, 90,000 cells/mL was added to 24-well plates with 1 mL of cell suspension and cultured for 24 h [24]. After complete cell fusion, they were treated with free FITC-dextran (F-dextran), FITC-dextran mixed with VB (mix solution), and FITC-dextran-labeled VB-ME (F-VB-ME) for 2 h at 37 °C. Subsequently, the cells were washed with 1× PBS and fixed with paraformaldehyde for 15 min, and all the wells were stained with 250 µL DAPI solution (Solarbio, Beijing, China, S2110) at room temperature for 10 min. Images were captured using fluorescence microscopy (Olympus, Tokyo, Japan), and relative fluorescence levels were calculated using ImageJ and normalized to the control groups.

### 2.3. Determination of Cell Viability by MTT Assay

LX-2 cells were plated at a density of 8000 cells/mL in 96-well microplates, with 100 μL of cells added to each well and allowed to grow until reaching confluence. Subsequently, the LX-2 cells were treated with varying concentrations (100, 50, 25, 12.5, 6.25, and 0 µM) of VB-ME and VB for 24 h. Following the treatment, the cells were incubated with 10 µL MTT solution (5 mg/mL, Aladdin, T100896-5 g) for an additional 4 h at 37 °C. After removing the medium, 110 µL DMSO per well was added, and the mixture was shaken for 10 min to ensure thorough mixing. The absorbance was then measured at 490 nm [22].

### 2.4. Reverse Transcription–Quantitative PCR (RT-qPCR)

Total RNA was extracted from LX-2 cells using TRIzol Reagent (Invitrogen, Life Technologies, Tokyo, Japan) following the manufacturer’s instructions. Subsequently, cDNA was synthesized from the total RNA using an RT kit (Promega, Madison, WI, USA) with 1 μg of total RNA as the template under the following conditions: 25 °C for 5 min, 45 °C for 60 min, and 70 °C for 15 min. The qPCR was performed using SYBR Green Supermix (Bio-rad, Hercules, CA, USA), and gene primer sequences were synthesized by Hongxun (Suzhou, China), as detailed in Supplementary Table S1. HPRT1 was used as the endogenous control gene to account for the mRNA expression levels of other genes in each sample. A statistical analysis was carried out using the ∆∆Ct method [25].

### 2.5. Protein Extraction and Western Blot Analysis

Protein lysate was prepared by adding 1% protease inhibitor PMSF in NP-40 buffer; 130 µL of protein lysate was added to each well and lysed on ice for 30 min. Centrifugation was performed at 4 °C, 12,000 rpm for 15 min. Protein supernatant was collected after centrifugation and quantified by BCA. After quantification, all protein samples were mixed with 5× loading buffer and boiled for 10 min. SDS polyacrylamide gel electrophoresis was performed by adding 15 µg of protein samples to each well and transferring the proteins to the NC membrane by wet transfer. The membrane was then closed with 5% skim milk powder for 1 h at room temperature. The membranes were incubated with specific antibodies diluted with TBST (TBS with 0.1% Tween20-buffered saline) overnight at 4 °C. After primary antibody incubation, the membrane was washed three times with TBST for 10 min each time, and then the secondary antibody (Anti-rabbit IgG HRP-linked Antibody, CST, Danvers, MA, USA, 7074S; Anti-mouse IgG,HRP-linked Antibody, CST, Danvers, MA, USA, 7076S) was incubated for 1 h at room temperature. Protein bands were visualized by chemiluminescence (ECL blotting reagent, Bio-Rad, Hercules, CA, USA, 1705061) [26]. The following antibodies were used for Western blotting: FN1 (1:1000, Abclonal, Wuhan, China, A12932) COL1A1 (1:1000, Abclonal, Wuhan, China, A1352), p38 (1:1000, Abmart, Shanghai, China, T55600), p-p38 (1:1000, Abmart, Shanghai, China, T40076), JNK (1:1000, CST, Danvers, MA, USA, 9252S), p-JNK(1:1000, CST, Danvers, MA, USA, 9255S), ERK(1:1000, Huabio, Hang Zhou, China, ET1601-29), p-ERK(1:1000, Huabio, Hang Zhou, China, ET1610-13), and β-tubulin (1:5000, Fdbio science, Hang Zhou, China, FD0064).

### 2.6. Construction of Pseudo-Ternary Phase Diagrams

Food-grade water-in-oil (W/O) microemulsions were prepared with isopropyl myristate (IPM) as the continuous phase, an absolute ethanol solution as the dispersed phase, and a blend of soy phosphatidylcholine (SoyPC) as the surfactants. The mixture of oil and surfactants was titrated with an aqueous phase while gently being turned upside down, until transparent microemulsions were formed [27]. The oil phase was initially blended with a surfactant mixture at weight ratios ranging from 1:9 to 9:1. The mixture was then titrated with water until the solubilization limit was reached, indicated by the appearance of turbidity. Simultaneously, the boundary of the single-phase region was determined by recording the proportion of the water phase.

### 2.7. Preparation of VB-Loaded Microemulsion

In this study, the dissolution procedure was employed, where a concentrated VB solution replaced distilled water and was added to the mixture solution of IPM and SoyPC as the dispersed phase [28]. The formation procedure was maintained at a constant temperature of 25 °C.

### 2.8. Particle Size, Size Distribution, and Zeta Potential Analysis

The microemulsion was analyzed for mean particle size, particle-size distribution (polydispersity index), and surface charge (zeta potential) using a Zetasizer Pro (Malvern Panalytical Technologies, Malvern, UK) [29]. All measurements were conducted at a temperature of 25 ± 1 °C.

### 2.9. Infrared Spectrum Analysis

Fourier-transform infrared spectroscopy (FTIR) was employed to analyze the VB raw materials [27], IPM, SoyPC, blank microemulsion, and the microemulsion containing 50 µM VB. The analysis was conducted in the range of 650–4000 cm^−1^ with a resolution of 2 cm^−1^ using an FTIR spectrometer (Cary630, Agilent Technologies, Palo Alto, CA, USA).

### 2.10. High-Performance Liquid Chromatography

HPLC analysis was conducted using an Agilent HPLC Spectra System with detection at a wavelength of 320 nm [17]. Separation was achieved via gradient elution on a 4.6 × 250 mm reverse-phase C-18 (5 μm) column. The elution was carried out using methanol (eluent A) and a mixture of water/acetic acid (95:5) (eluent B). The gradient profile was set as follows: 85–60% B (0–25 min), 60% B (25–30 min), 60–37% B (30–45 min), 37% B (45–47 min), and 37–0% B (47–52 min). The flow rate was maintained at 1 mL/min, and the sample volume injected was 20 μL.

### 2.11. Cell Migration Assay

For the scratch-wounding cell migration assay, cells were seeded on chamber slides at a density of 80,000 cells per well in 24-well plates with 1% FBS/DMEM and grown until reaching 100% confluence (12 h). An artificial wound was created by scratching the bottom of the plate with a 20 µL pipette tip. Immediately after creating the wound, detached cells and debris were removed with three washes of PBS. The cells were then pretreated with 50 µM VB or 50 µM VB-ME for 6 h, followed by treatment with 5 ng/mL TGFβ1 for 24 h. Photographs of the cells were taken before (0 h) and 24 h after TGFβ1 treatment using an inverted phase-contrast microscope (Olympus, Tokyo, Japan). The cell migration rate was calculated as (X_0h_ − X_24h_)/X_24h_, where X_0h_ and X_24h_ represent the widths of the scratch at 0 h and 24 h after exposure, respectively [30]. Relative migration levels were calculated using ImageJ and normalized to the control groups.

### 2.12. Ki67 Immunofluorescence Assay

The Ki67 immunofluorescence assay was employed to study the effect of the compounds on the proliferation of LX-2 cells [22]. Cells were seeded in 24-well plates at a density of 20,000 cells per well with 1% FBS/DMEM. After 12 h, the cells were pretreated with 50 µM VB or VB-ME for 6 h and then treated with 5 ng/mL TGFβ1 for 24 h. At room temperature, the cells were fixed with 4% PFA for 10 min and then permeabilized with 0.1% TritonX-100 diluted with PBS for 10 min. Subsequently, they were blocked with 3% horse serum at room temperature for 1 h. The primary antibodies (Ki67, 1:100, Thermo, Waltham, MA, USA, 11-5698-80) were diluted in 3% horse serum at 300 µL per well and then incubated overnight at 4 °C. The secondary antibodies were incubated at room temperature for 2 h. All wells were stained with 250 µL DAPI solution (Solarbio, Beijing, China, S2110) at room temperature for 10 min, and images were captured using fluorescence microscopy (Olympus, Tokyo, Japan). Relative fluorescence levels were calculated using ImageJ 1.8.0 and normalized to the control groups.

### 2.13. Reactive Oxygen Species (ROS) Assay

The evaluation of intracellular oxidants was conducted using the fluorescent probe 2′,7′-dichlorodihydrofluorescein diacetate (DCFH-DA; MedChemExpress, Monmouth Junction, NJ, USA, HY-D0940) [31,32]. This cell-permeable, non-fluorescent probe undergoes de-esterification intracellularly, transforming into highly fluorescent 2′,7′-dichlorofluorescein upon oxidation. The cells were pretreated with 50 µM VB or VB-ME for 6 h and then treated with TGFβ1 for 24 h. After 24 h, the cells were stained with Hoechst 33,342 and washed with PBS. Subsequently, they were incubated with a solution containing 20 µM DCFH-DA for 30 min at 37 °C, followed by three washes with PBS. After staining, immediate observation was conducted with a fluorescence microscope (Olympus, Tokyo, Japan), and photographs were taken. The relative level of intracellular ROS was quantified using ImageJ 1.8.0 and normalized to the control group.

### 2.14. Detecting the Activity of SOD

Superoxide dismutase (SOD) levels were assayed using a Total SOD Assay Kit (Beyotime, Haimen, China, S0101). Briefly, cells were washed once with PBS at 4 °C, and an appropriate SOD sample preparation solution was added to fully lyse the cells. The cell lysate was then centrifuged at 600× *g* for 10 min at 4 °C using a cryo-centrifuge. The supernatant was collected and used for the determination of SOD enzyme activity, which was expressed as activity units per mg of protein [33].

### 2.15. Statistical Analysis

A statistical analysis was conducted using GraphPad Prism 8.0. One-way ANOVA analyses were employed to determine statistical significance. All numerical results are presented as the mean ± standard error of the mean (SEM). A *p*-value < 0.05 indicates significance.

## 3. Results

### 3.1. VB Suppresses the mRNA and Protein Levels of Fibrogenic Genes in Activated LX-2 Cells

**Figure 1:**
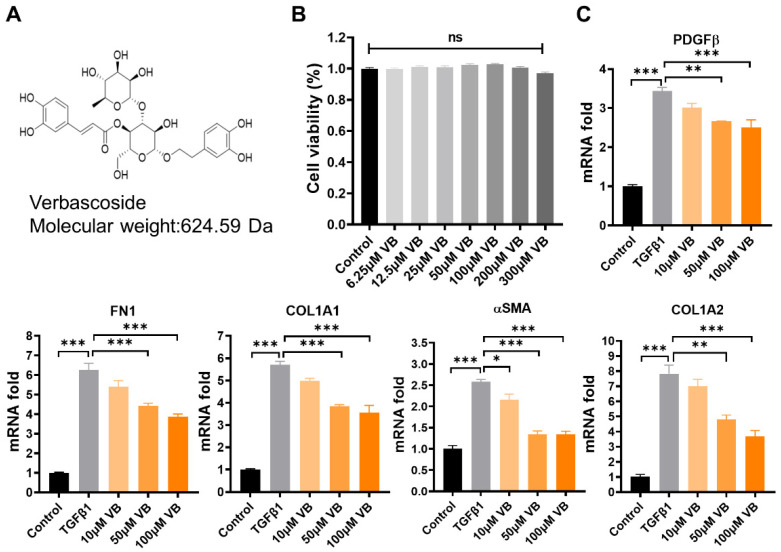
VB suppresses mRNA levels of fibrogenic genes in activated LX-2 cells. (**A**) Chemical information of VB. (**B**) LX-2 cells were treated with VB at concentrations of 3.125, 6.25, 12.5, 25, 50, and 100 µM for 24 h, and cell viability was assessed using the MTT assay. The control group was treated with PBS. The data are presented as mean ± SEM (*n* = 4), with ‘ns’ indicating no significant differences. (**C**) LX-2 cells were pretreated with VB at concentrations of 0, 50, or 100 μM for 6 h, followed by treatment with TGFβ1 (5 ng/mL) for 24 h. mRNA expression levels of fibrogenic genes, including αSMA, COL1A1, COL1A2, FN1, and PDGFβ, were measured using qPCR. Inactive LX-2 cells served as the control group. The data presented are representative of three independent replicate experiments. Data are presented as mean ± SEM (*n* = 3). *: *p* < 0.05, **: *p* < 0.01, ***: *p* < 0.001.

**Figure 2:**
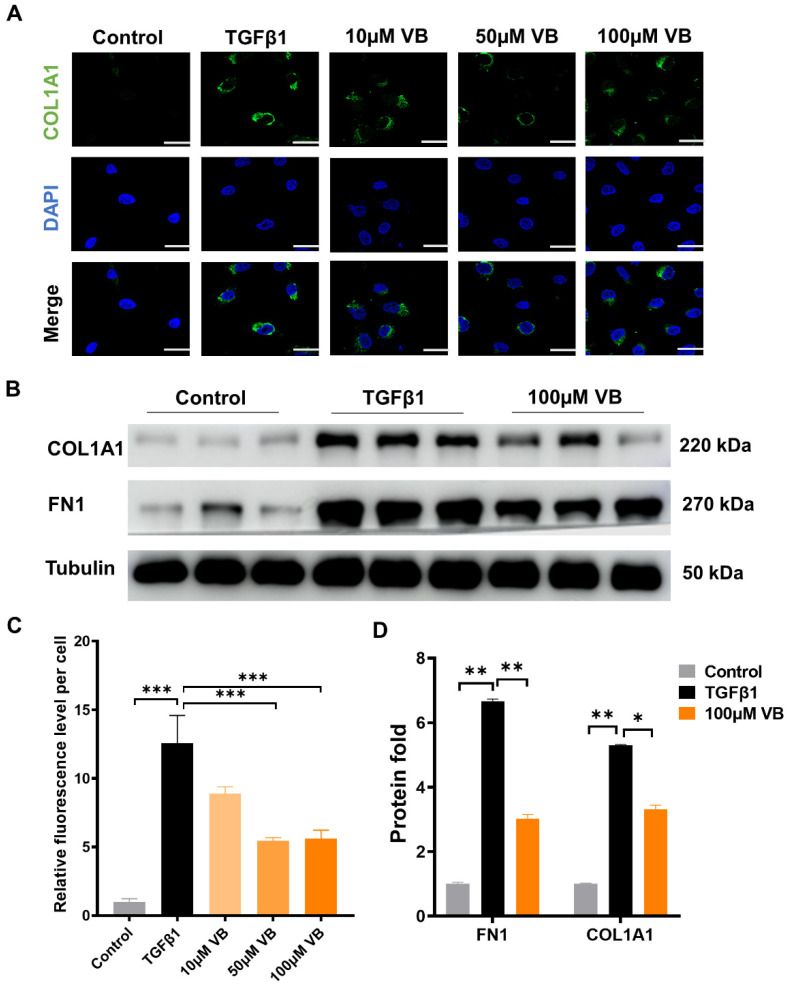
VB inhibits the protein expression of fibrogenic genes at the protein level. LX-2 cells were pretreated with VB at different concentrations for 6 h, followed by treatment with TGFβ1 (5 ng/mL) for 24 h. Inactive LX-2 cells served as the control group. (**A**) Confocal images of LX-2 cells stained with COL1A1 antibody and DAPI. Scale bar, 20 μm. (**B**) Western blot assay images depicting protein levels of fibrogenic genes, including COL1A1 and FN1, in LX-2 cells. (**C**) Quantitative analysis of fluorescence levels in (**A**). (**D**) Quantitative analysis of the bands of Western blot in (**C**). The data presented are representative of three independent replicate experiments. Data are presented as mean ± SEM (*n* = 3). *: *p* < 0.05, **: *p* < 0.01, ***: *p* < 0.001.

Verbascoside (VB) was isolated from whole plant samples of Pedicularis rex C. B. Clark, and its chemical structure information (Figure 1A) was identified using 1H-NMR and 13C-NMR, referring to the previously reported methods [11,28] (Supplementary Figures S1 and S2). The purity, determined to be above 95% by 1H-NMR and confirmed by the HPLC method, ensured the quality of the sample. LX-2 cells, a valuable human hepatic stellate cell line, were used to analyze the effects of VB on hepatic fibrosis [34]. First, the cytotoxicity of VB on LX-2 cells was assessed using the MTT assay (Figure 1B). The cell viability showed no significant difference among the groups treated with VB at concentrations ranging from 6.25 to 300 μM compared to the control group. Within this safe range, concentrations of 10 µM, 50 µM, and 100 µM of VB were used to treat LX-2 cells activated by TGFβ1. The elevated mRNA levels of fibrogenic genes in the activated LX-2 cells, including αSMA, PDGFβ, COL1A2, FN1, and COL1A1, were significantly suppressed by treatment with 50 µM or 100 µM of VB for 24 h (Figure 1C). Immunofluorescence and Western blot assays were further employed to examine the effects of VB on the protein levels of fibrogenic genes (Figure 2A–D). Similar to the mRNA results, the elevated protein levels of fibrogenic genes such as COL1A1 and FN1 were significantly suppressed in activated LX-2 cells treated with VB. These findings indicate that VB is potent in suppressing HSC activation.

### 3.2. VB Scavenges ROS in Activated LX-2 Cells and Downregulates MAPK Pathway

**Figure 3:**
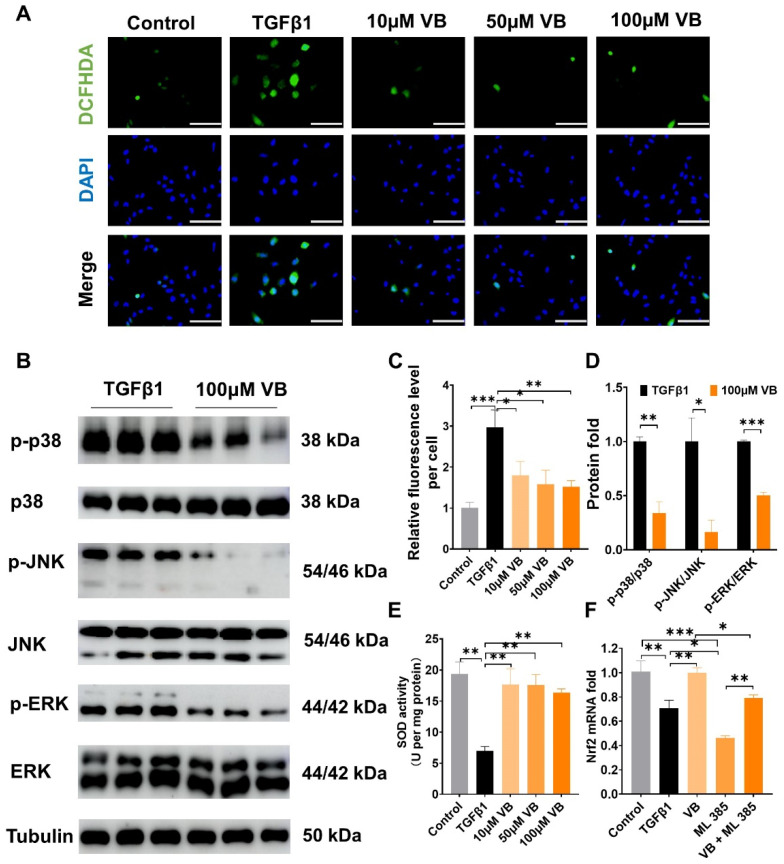
VB scavenges ROS in activated LX-2 cells and downregulates the MAPK pathway. (**A**) LX-2 cells were pretreated with VB at different concentrations for 6 h, followed by treatment with TGFβ1 (5 ng/mL) for 24 h. Inactive LX-2 cells served as the control group. Intracellular ROS levels were determined using the DCFH-DA assay. Green represents intracellular ROS, and blue represents the cell nucleus. Scale bar, 100 µM. (**B**) Western blot assay images depicting protein levels of p38, JNK, and ERK. (**C**) The fluorescence intensity of DCFH-DA was normalized to the control group. (**D**) Quantitative analysis of the bands of Western blot in (**B**). (**E**) Intracellular SOD activities were determined using a superoxide dismutase assay kit. (**F**) The mRNA levels of Nrf2 in LX-2 cells were measured under different treatment conditions. LX-2 cells were treated as described in (**A**). In the VB group, cells were treated with 100 μM VB; in the ML385 group, cells were treated with 5 μM ML385; in the VB + ML385 group, cells were treated with 100 μM VB and 5 μM ML385. The data presented are representative of three independent replicate experiments. Data are presented as mean ± SEM (*n* = 3), *: *p* < 0.05, **: *p* < 0.01, ***: *p* < 0.001.

Reactive oxygen species (ROS) play an important role in directly activating HSCs [35]. VB, with its aromatic nucleus and multiple −OH groups, has been shown to reduce reactive species in other diseases [12,36]. We investigated the ROS-scavenging capability of VB in LX-2 cells using the fluorescent probe DCFH-DA [32]. VB significantly reduced the elevated ROS levels in activated LX-2 cells at concentrations of 10, 50, and 100 µM (Figure 3A,C). Furthermore, we examined VB’s regulation of the MAPK pathway, a critical signaling crossroad sustaining liver fibrosis in conjunction with ROS generation. VB had no effect on the total protein levels of p38, JNK, and ERK, but it downregulated their phosphorylation levels (Figure 3B,D). These results demonstrate that VB suppresses the expression of fibrogenic genes in activated LX-2 cells by downregulating the MAPK pathway. To further analyze the exact mechanism of ROS scavenging by VB, we assessed its effects on the activity of the antioxidant enzyme SOD. Our findings indicated that VB significantly increased SOD levels at concentrations of 10, 50, and 100 μM compared to the TGF-β treatment group (Figure 3E). This result aligns with the observed reduction in ROS levels induced by VB. Subsequently, we evaluated the influence of VB on the protein expression levels of Nrf2 using an immunofluorescent assay (Supplementary Figure S3). The TGF-β treatment inhibited Nrf2 levels in activated LX-2 cells, but this inhibition was significantly reversed by VB treatment. To further investigate the specific regulation of Nrf2 expression by VB, we used ML385, a Nrf2 inhibitor, in combination with TGF-β to treat LX-2 cells. ML385 further decreased Nrf2 mRNA levels compared to the TGF-β treatment group (Figure 3F). However, VB treatment effectively counteracted their inhibition on Nrf2 mNRA levels. These findings suggest that VB scavenges ROS by increasing SOD activity and regulating the MAPK and Nrf2 pathways.

### 3.3. Development and Characterization of Verbascoside-Loaded Reverse Microemulsion

**Figure 4:**
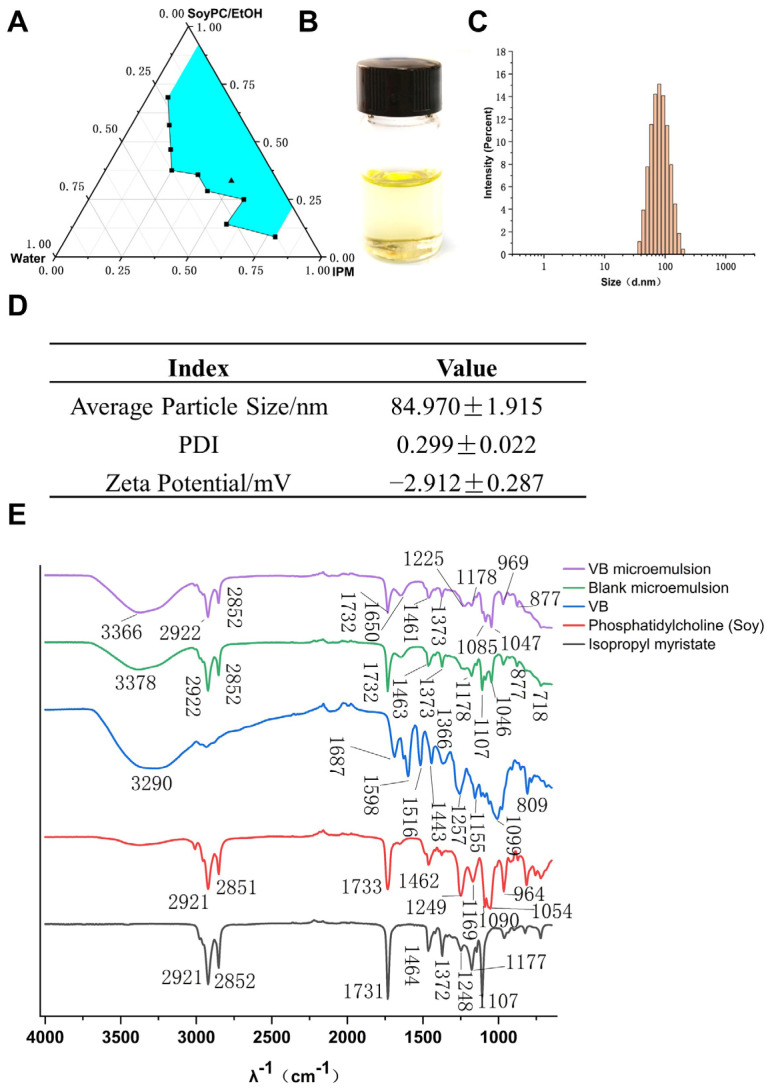
Development and characterization of VB-ME. (**A**) Pseudo-ternary phase diagram of the microemulsion system: IPM/Water/SoyPC in EtOH at 25 °C. IPM: isopropyl myristate, SoyPC: soy phosphatidylcholine, EtOH: ethanol. Blue area presents microemulsion zone. (**B**) The visual image of VB-ME. (**C**) The droplet-size distribution of VB-ME. (**D**) The averaged hydrodynamic diameter, PDI, and zeta potential of VB-ME. (**E**) The infrared spectrograms of isopropyl myristate, phosphatidylcholine (soy), VB, blank microemulsion, and VB microemulsion. The data presented are representative of three independent replicate experiments.

VB is a hydrophilic molecule with a molecular weight of 624.592. It has a high solubility and low permeability, categorizing it under class III in the Biopharmaceutical Classification System (BCS) [37]. Consequently, we developed a reverse microemulsion drug carrier system with the aim of enhancing the cellular uptake of VB. A biocompatible microemulsion system, comprising isopropyl myristate (IPM) as the oil solvent, soybean phosphatidylcholine (SoyPC) as the emulsifier, and ethanol as the co-emulsifier, was chosen to fabricate a pseudo-ternary phase diagram, as shown in Figure 4A. A superior microemulsion system was identified in this study, characterized by its composition of 49.5% IPM, 33.1% SoyPC in ethanol, and 17.4% water. This optimal formulation is denoted by a black triangle in the representation. Next, we loaded VB in the water phase and obtained a VB-loaded microemulsion (VB-ME) with a drug content of 7.56 mg/g. The visual appearance of VB-ME was clarity and transparency due to the thermodynamic stability of the microemulsion (Figure 4B). The droplet-size distribution of VB-ME was a normal distribution with a unimodal and narrow peak (Figure 4C). The averaged hydrodynamic diameter of VB-ME is 84.97 nm, and the zeta potential is −2.912 mV (Figure 4D). The slightly negative zeta potential and small droplet size of VB-ME would benefit cell membrane permeability [38].

FTIR is further employed to confirm the components in the VB-loaded microemulsion [39]. As shown in Figure 4E, the VB microemulsion exhibits characteristic peaks at 1732, 2922, and 2852 cm^−1^, corresponding to the ester and alkyl groups of IPM and SoyPC. A broad peak at 3366 cm^−1^ corresponds to the hydroxyl groups. Additionally, the peak at 1650 cm^−1^ is characteristic of the aromatic ring in VB. These data support the successful encapsulation of VB in the microemulsion system.

### 3.4. VB-ME Enhanced Stability and Cell Uptake of VB

**Figure 5:**
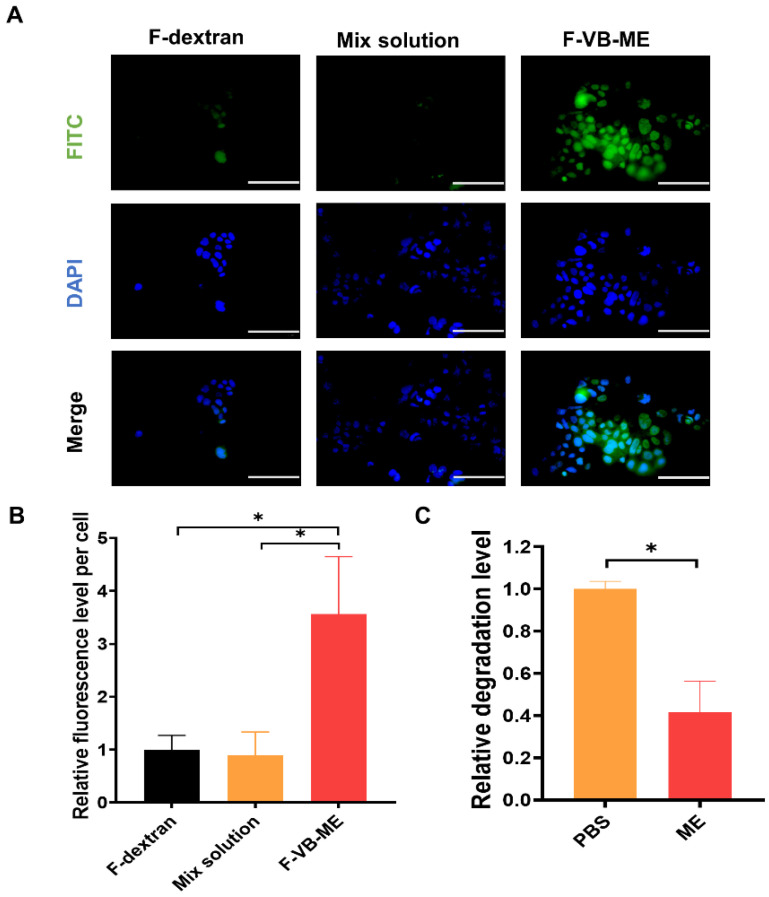
VB-ME increased cellular uptake in Caco-2 cells. (**A**) Caco2 cells were treated with FITC-dextran (F-dextran), FITC-dextran mixed with VB solution (mix group), or F-dextran co-loaded with VB-ME (F-VB-ME group) for 2 h. Fluorescence signals were observed using a fluorescence microscope. Scale bar, 100 µM. (**B**) The fluorescence intensity was normalized to the F-dextran group. (**C**) The stability of VB-ME and VB in PBS (pH 7.4) solution at 37 °C for 24 h. The relative degradation level was tested using the HPLC method. The data presented are representative of three independent replicate experiments. Data are presented as mean ± SEM (*n* = 5), *: *p* < 0.05.

VB has been reported to be unstable at pH 7.4. However, after encapsulation into a reverse microemulsion, the stability of VB was significantly enhanced (Figure 5C). This improvement is likely due to the bound status of water within the reverse microemulsion. The binding of water with IPM and other microemulsion excipients can reduce the degradation of VB in aqueous environments. The cellular uptake of VB solution and VB-ME was assessed using CLSM in Caco-2 cells. FITC-labeled dextran (F-dextran), belonging to the same BCS class III as VB, was co-loaded into VB-ME as a tracking molecule [24]. The cells were treated with free FITC-labeled dextran (F-dextran), a mixture of F-dextran and VB solution (mix solution), and F-dextran-co-loaded VB-ME (F-VB-ME) for 2 h. Both the F-dextran and mix solution groups showed minimal fluorescence signals. In contrast, the F-VB-ME group exhibited a noticeable fluorescence signal, with an intensity much higher than that of both the F-dextran and mix solution groups (Figure 5A,B). These results indicate that the microemulsion can significantly increase the cellular uptake rate of VB.

### 3.5. VB-ME Enhanced the Suppression of Fibrogenic Genes and Activation Features in LX-2 Cells

**Figure 6:**
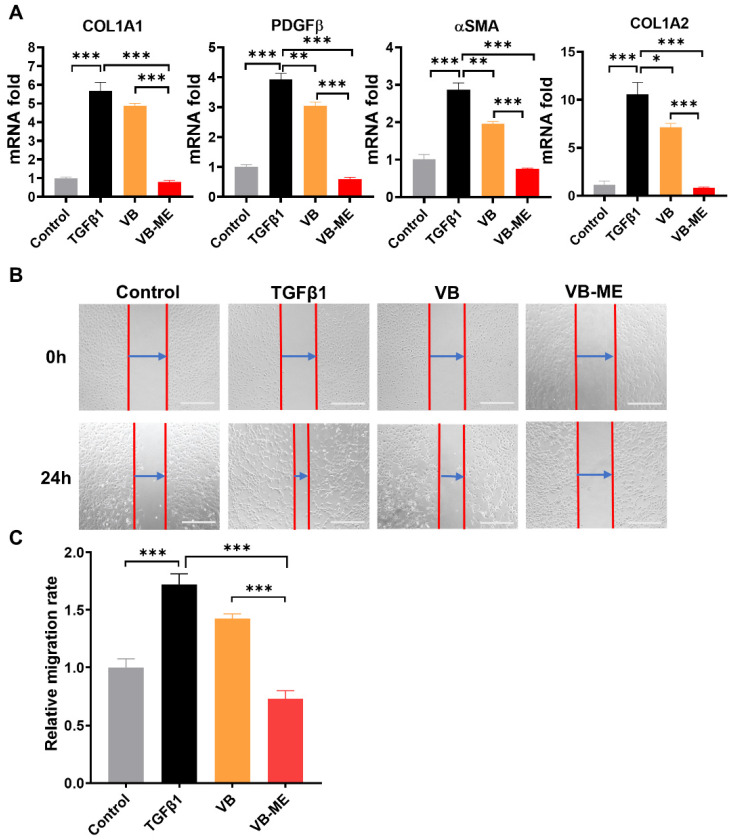
VB-ME enhanced the suppression of fibrogenic gene expression and migration in activated LX-2 cells. LX-2 cells were pretreated with either VB or VB-ME at a concentration of 50 μM VB for 6 h, followed by treatment with TGFβ1 (5 ng/mL) for 24 h. (**A**) The mRNA expression levels of fibrogenic genes, including αSMA, COL1A1, COL1A2, and PDGFβ, were measured using the qPCR method. Inactive LX-2 cells served as the control group. The data presented are representative of three independent replicate experiments. Data are presented as mean ± SEM (*n* = 3). *: *p* < 0.05, **: *p* < 0.01, ***: *p* < 0.001. (**B**) A scratch wound healing assay was performed after the same treatment process as in the previous figure. The red line indicates the scratched cell edge, and the blue arrow shows the range of cell migration. Scale bar, 500 µM. (**C**) The cell migration level was calculated as (Range (0 h) − Range (12 h))/Range (0 h), and the result was normalized to the TGFβ-treated group. The data presented are representative of three independent replicate experiments. Data are presented as mean ± SEM (*n* = 4), ***: *p* < 0.001.

Firstly, the cytotoxicity of VB-ME on LX-2 cells was assessed using the MTT assay (Supplementary Figure S4). The cell viability showed no significant difference among the groups treated with VB-ME at concentrations of VB in the range of 0 to 200 μM for both 24 h and 48 h. This indicates that the compositions of the microemulsion system did not induce additional toxicity to LX-2 cells. In activated LX-2 cell, VB-ME significantly enhanced the repression of fibrogenic gene expression compared to the same concentration of VB solution (Figure 6A). VB-ME significantly reduced the mRNA levels of αSMA, COL1A1, COL1A2, and PDGFβ to levels comparable to those in inactive LX-2 cells. These findings support the notion that VB-ME enhances the suppression of fibrogenic gene expression in activated LX-2 cells compared to VB solution.

**Figure 7:**
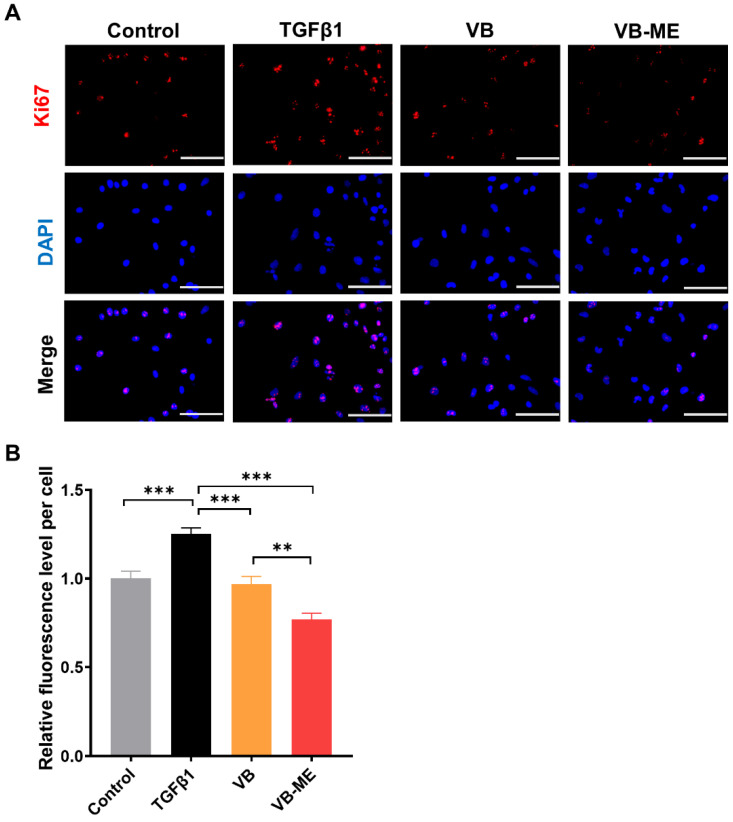
VB-ME enhanced the inhibition of proliferation in activated LX-2 cells. (**A**) Following the same treatment process as in the previous figure, cell proliferation levels were assessed using the Ki67 assay. Ki67 protein is shown in red, and nuclear staining with DAPI is in blue. Scale bar, 100 µM. (**B**) The relative proliferation rate was calculated by dividing the number of Ki67-positive cells by the total cell count, and the result was normalized to the TGFβ-treated group. The data presented are representative of three independent replicate experiments. Data are presented as mean ± SEM (*n* = 10), **: *p* < 0.01, ***: *p* < 0.001.

Wound-healing ability is a crucial characteristic of activated HSCs with high migration rates [40]. After 24 h of wound healing, TGFβ-induced activated LX-2 cells exhibited a much higher migration level than the control group of inactive LX-2 cells (Figure 6B,C). However, the migration level was reduced by treatment with VB. VB-ME significantly decreased the migration levels compared to the TGFβ-induced group. Another feature of activated HSCs is high proliferation. The Ki67 assay was conducted to evaluate the proliferation of LX-2 cells after treatment (Figure 7A,B). Similarly, the TGFβ-induced high proliferation of activated LX-2 cells was significantly inhibited by VB, and the inhibitory effect was further enhanced by VB-ME. These results indicate that VB-ME was able to enhance the inhibition of the two crucial features of the migration and proliferation of activated HSCs.

### 3.6. VB-ME Enhanced the Scavenging of ROS in LX-2 Cells

**Figure 8:**
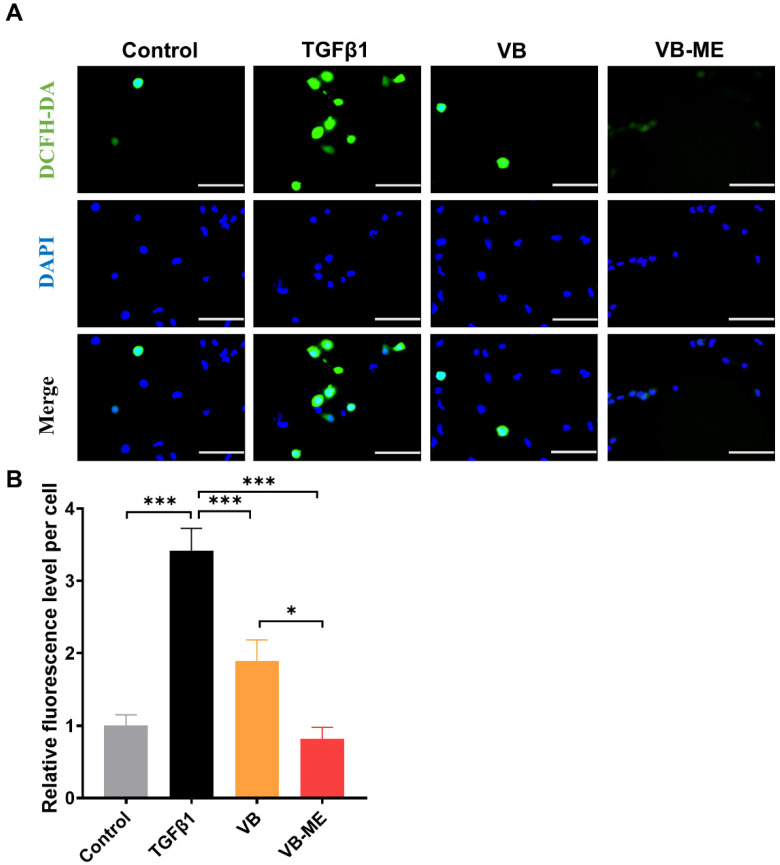
VB-ME enhanced the scavenging of ROS in LX-2 cells. LX-2 cells were pretreated with VB at different concentrations for 6 h, followed by treatment with TGFβ1 (5 ng/mL) for 24 h. Inactive LX-2 cells served as the control group. (**A**) Intracellular ROS levels were determined using the DCFH-DA assay. Green represents intracellular ROS, and blue represents the cell nucleus. Scale bar, 100 µM. (**B**) The fluorescence intensity of DCFH-DA was normalized to the control group. The data presented are representative of three independent replicate experiments. Data are presented as mean ± SEM (*n* = 5), *: *p* < 0.05, ***: *p* < 0.001.

Previously, we showed that VB can scavenge ROS and downregulate the MAPK pathway to suppress HSC activation. We further examined the enhanced effects of VB-ME on ROS scavenging. Following TGFβ-induced activation, the ROS level in LX-2 cells increased approximately 3.5-fold compared to the control group (Figure 8). However, treatment with VB led to an approximate 1.5-fold reduction in ROS levels, while treatment with VB-ME reduced ROS levels to those observed in inactivated LX-2 cells. These results indicate that VB-ME can efficiently enhance the ROS-scavenging ability of VB.

## 4. Discussion

The activation of HSCs into proliferative, fibrogenic myofibroblasts is well established as the central driver of liver fibrosis in both preclinical and clinical studies [41]. Extensive research has focused on investigating the regulation of in vitro HSC activation to find potential treatments for liver fibrosis [7,9]. Among these studies, the LX-2 cell line is an ideal tool for evaluating drugs that target HSC activation, as it can replicate the in vivo phenotype of human HSCs and elucidate pathways involved in human liver fibrosis [34]. TGF-β1, a known stimulator of HSC activation, was used to treat LX-2 cells to create an in vitro liver fibrogenic model [42,43]. In this study, we found that VB at concentrations of 50 µM and 100 µM significantly suppressed the elevated expression of α-SMA, a reliable marker for HSC activation, in LX-2 cells activated by TGF-β1. Additionally, the increased levels of PDGFβ, a potent mitogen for HSCs, were significantly suppressed by VB treatment. Moreover, the elevated extracellular matrix (ECM) proteins, such as COL1A1, COL1A2, and FN1, in activated LX-2 cells were efficiently suppressed by VB treatment. These results support the potential medical application of VB in ameliorating liver fibrosis.

ROS can act as both an inducer and effector of the TGF-β signaling pathway, generating a vicious cycle for liver fibrosis and HSC activation [44]. In our studies, we observed that ROS levels were elevated in activated LX-2 cells induced by TGF-β1, while VB effectively scavenged the ROS. To elucidate the underlying mechanism, we investigated the impact of VB on the MAPK cascade pathway, which is activated by ROS generation in HSCs [45]. The MAPK cascades, encompassing ERK, JNK, and p38, are known to respond to various cellular stressors [46]. ROS-induced oxidative stress has been widely recognized as an activator of MAPK pathways in several cell types, including human HSCs [47]. In our study, we found consistent results indicating that both ROS levels and the MAPK cascade pathway in activated LX-2 cells were downregulated by VB treatment. These parallel effects might suggest that VB’s ROS-scavenging mechanism involves the inhibition of the MAPK cascade pathway. Additional experiments will be necessary to demonstrate the causative link between ROS scavenging and MAPK inhibition. Additionally, VB treatment increased the levels of the antioxidant enzyme SOD and upregulated the expression of Nrf2, the transcriptional master regulator of cellular responses to oxidative stress. These findings support the notion that VB mitigates HSC activation by scavenging ROS, offering a potential therapeutic avenue for targeting HSC-mediated fibrosis.

The oil phase comprising IPM and the surfactant SoyPC used in our microemulsion formulation is known to be biosafe [48,49], as corroborated by cytotoxicity data from this study. Encapsulating VB within this water–oil system effectively shields it from degradation, as the water within the optimal VB-ME formulation remains in a bound state at the interface, thereby enhancing VB stability under neutral pH conditions [50]. Specifically, VB’s stability in VB-ME was found to increase nearly tenfold compared to that in pH 7.4 PBS solution. Moreover, the reverse microemulsion system is anticipated to enhance VB’s transmembrane permeability due to the biocompatibility of the IPM oil phase with the cell membrane [51,52]. This assertion is supported by the significantly improved cellular uptake of VB-ME observed in Caco-2 cells. Furthermore, VB-ME demonstrated a markedly increased suppression of fibrogenic genes and activation-related features such as migration and proliferation in LX-2 cells compared to VB solution alone. These findings collectively suggest that VB-ME enhances VB’s inhibitory effects on HSC activation by increasing cellular uptake and stabilizing VB.

## 5. Conclusions

In this study, we demonstrated that VB suppresses HSC activation by scavenging ROS and downregulating the MAPK pathway. The efficacy of VB was significantly enhanced by encapsulating it into a biosafe reverse microemulsion, VB-ME. Serving as a simple and effective carrier, VB-ME is a promising therapeutic approach for liver fibrosis, providing a novel and effective carrier to enhance VB’s anti-fibrotic properties.

## Data Availability

Data are contained within the article and Supplementary Materials.

## Supplementary Materials

The following supporting information can be downloaded at: https://www.mdpi.com/article/10.3390/antiox13080907/s1. Figure S1: ^1^H-NMR of Verbascoside (CD3OD). Figure S2: ^13^C-NMR of Verbascoside (CD3OD). Figure S3: The expression levels of Nrf2 protein in LX-2 cells. Figure S4: Cell viability assessed by the MTT assay. Table S1: Primers for RT-qPCR.

## References

[1] Devarbhavi H., Asrani S.K., Arab J.P., Nartey Y.A., Pose E., Kamath P.S. (2023). Global burden of liver disease: 2023 update. J. Hepatol..

[2] Orci L.A., Sanduzzi-Zamparelli M., Caballol B., Sapena V., Colucci N., Torres F., Bruix J., Reig M., Toso C. (2022). Incidence of hepatocellular carcinoma in patients with nonalcoholic fatty liver disease: A systematic review, meta-analysis, and meta-regression. Clin. Gastroenterol. Hepatol..

[3] Fallowfield J., Hayes P. (2011). Pathogenesis and treatment of hepatic fibrosis: Is cirrhosis reversible?. Clin. Med..

[4] Puche J.E., Saiman Y., Friedman S.L. (2013). Hepatic stellate cells and liver fibrosis. Compr. Physiol..

[5] Lee U.E., Friedman S.L. (2011). Mechanisms of hepatic fibrogenesis. Best Pract. Res. Clin. Gastroenterol..

[6] Baghaei K., Mazhari S., Tokhanbigli S., Parsamanesh G., Alavifard H., Schaafsma D., Ghavami S. (2022). Therapeutic potential of targeting regulatory mechanisms of hepatic stellate cell activation in liver fibrosis. Drug Discov. Today.

[7] Higashi T., Friedman S.L., Hoshida Y. (2017). Hepatic stellate cells as key target in liver fibrosis. Adv. Drug Deliv. Rev..

[8] Wei Q., Guo J.S. (2022). Developing natural marine products for treating liver diseases. World J. Clin. Cases.

[9] Chan Y.T., Wang N., Tan H.Y., Li S., Feng Y. (2020). Targeting hepatic stellate cells for the treatment of liver fibrosis by natural products: Is it the dawning of a new era?. Front. Pharmacol..

[10] Shan L., Liu Z., Ci L., Shuai C., Lv X., Li J. (2019). Research progress on the anti-hepatic fibrosis action and mechanism of natural products. Int. Immunopharmacol..

[11] Rossi R., Mainardi E., Vizzarri F., Corino C. (2023). Verbascoside-rich plant extracts in animal nutrition. Antioxidants.

[12] Sciandra F., Bottoni P., De Leo M., Braca A., Brancaccio A., Bozzi M. (2023). Verbascoside elicits its beneficial effects by enhancing mitochondrial spare respiratory capacity and the nrf2/ho-1 mediated antioxidant system in a murine skeletal muscle cell line. Int. J. Mol. Sci..

[13] Zhao Y., Wang S., Pan J., Ma K. (2023). Verbascoside: A neuroprotective phenylethanoid glycosides with anti-depressive properties. Phytomedicine.

[14] Mao Y., Yu J., Da J., Yu F., Zha Y. (2023). Acteoside alleviates uuo-induced inflammation and fibrosis by regulating the hmgn1/tlr4/trem1 signaling pathway. PeerJ.

[15] Jiang Y., Lin X., Mao Y., Zhao J., Zhang G., Yu J., Dong R., Zha Y. (2022). Acteoside alleviates renal fibrosis by inhibiting β-catenin/ctgf signaling pathway in uuo rats. Nat. Prod. Commun..

[16] Chen C.Y., Tung H.Y., Tseng Y.F., Huang J.S., Shi L.S., Ye Y.L. (2022). Verbascoside and isoverbascoside ameliorate transforming growth factor β1-induced collagen expression by lung fibroblasts through Smad/non-Smad signaling pathways. Life Sci..

[17] D’Imperio M., Cardinali A., D’Antuono I., Linsalata V., Minervini F., Redan B.W., Ferruzzi M.G. (2014). Stability–activity of verbascoside, a known antioxidant compound, at different pH conditions. Food Res. Int..

[18] Xiao Y., Ren Q., Wu L. (2022). The pharmacokinetic property and pharmacological activity of acteoside: A review. Biomed. Pharmacother..

[19] Zhang W., Huo S.X., Wen Y.L., Xing H., Zhang Q., Li N., Zhao D., Sun X.L., Xu J., Yan M. (2015). Pharmacokinetics of acteoside following single dose intragastric and intravenous administrations in dogs. Chin. J. Nat. Med..

[20] Yang X., Li W., Li S., Chen S., Hu Z., He Z., Zhu X., Niu X., Zhou X., Li H. (2024). Fish oil-based microemulsion can efficiently deliver oral peptide blocking pd-1/pd-l1 and simultaneously induce ferroptosis for cancer immunotherapy. J. Control. Release.

[21] Nikolaev B., Yakovleva L., Fedorov V., Li H., Gao H., Shevtsov M. (2023). Nano- and microemulsions in biomedicine: From theory to practice. Pharmaceutics.

[22] Yuan X., Yang J., Huang Y., Li J., Li Y. (2023). Gut microbiota metabolite 3-indolepropionic acid directly activates hepatic stellate cells by ros/jnk/p38 signaling pathways. Biomolecules.

[23] Xu S., Chen Y., Miao J., Li Y., Liu J., Zhang J., Liang J., Chen S., Hou S. (2024). Esculin inhibits hepatic stellate cell activation and ccl(4)-induced liver fibrosis by activating the nrf2/gpx4 signaling pathway. Phytomedicine.

[24] Zheng Y., Xu G., Ni Q., Wang Y., Gao Q., Zhang Y. (2022). Microemulsion delivery system improves cellular uptake of genipin and its protective effect against aβ1-42-induced pc12 cell cytotoxicity. Pharmaceutics.

[25] Xue W., Zender L., Miething C., Dickins R.A., Hernando E., Krizhanovsky V., Cordon-Cardo C., Lowe S.W. (2007). Senescence and tumour clearance is triggered by p53 restoration in murine liver carcinomas. Nature.

[26] Shen Y., Yang X., Dong N., Xie X., Bai X., Shi Y. (2007). Generation and selection of immunized fab phage display library against human b cell lymphoma. Cell Res..

[27] Zhu S., Zeng J., Pan C., Chai Y., Bai M., Li J., Chen A. (2023). Reverse microemulsions as nano-carriers of tea polyphenols retard oxidation of eucommia ulmoides oliver seed oil. Colloids Surf. A Physicochem. Eng. Asp..

[28] He E., Li H., Li X., Wu X., Lei K., Diao Y. (2022). Transdermal delivery of indirubin-loaded microemulsion gel: Preparation, characterization and anti-psoriatic activity. Int. J. Mol. Sci..

[29] He J., Zhu Q., Dong X., Pan H., Chen J., Zheng Z.P. (2017). Oxyresveratrol and ascorbic acid o/w microemulsion: Preparation, characterization, anti-isomerization and potential application as antibrowning agent on fresh-cut lotus root slices. Food Chem..

[30] Cao X., Liang Y., Liu R., Zao X., Zhang J., Chen G., Liu R., Chen H., He Y., Zhang J. (2022). Uncovering the pharmacological mechanisms of gexia-zhuyu formula (gxzy) in treating liver cirrhosis by an integrative pharmacology strategy. Front. Pharmacol..

[31] Gabbia D., Carpi S., Sarcognato S., Cannella L., Colognesi M., Scaffidi M., Polini B., Digiacomo M., Esposito Salsano J., Manera C. (2021). The extra virgin olive oil polyphenol oleocanthal exerts antifibrotic effects in the liver. Front. Nutr..

[32] Kim H., Xue X. (2020). Detection of total reactive oxygen species in adherent cells by 2′,7′-dichlorodihydrofluorescein diacetate staining. J. Vis. Exp..

[33] Weng Q., Sun H., Fang C., Xia F., Liao H., Lee J., Wang J., Xie A., Ren J., Guo X. (2021). Catalytic activity tunable ceria nanoparticles prevent chemotherapy-induced acute kidney injury without interference with chemotherapeutics. Nat. Commun..

[34] Xu L., Hui A.Y., Albanis E., Arthur M.J., O’Byrne S.M., Blaner W.S., Mukherjee P., Friedman S.L., Eng F.J. (2005). Human hepatic stellate cell lines, lx-1 and lx-2: New tools for analysis of hepatic fibrosis. Gut.

[35] Ramos-Tovar E., Muriel P. (2020). Molecular mechanisms that link oxidative stress, inflammation, and fibrosis in the liver. Antioxidants.

[36] Burgos C., Muñoz-Mingarro D., Navarro I., Martín-Cordero C., Acero N. (2020). Neuroprotective potential of verbascoside isolated from acanthus mollis l. Leaves through its enzymatic inhibition and free radical scavenging ability. Antioxidants.

[37] Papich M.G., Martinez M.N. (2015). Applying biopharmaceutical classification system (bcs) criteria to predict oral absorption of drugs in dogs: Challenges and pitfalls. AAPS J..

[38] Yameen B., Choi W.I., Vilos C., Swami A., Shi J., Farokhzad O.C. (2014). Insight into nanoparticle cellular uptake and intracellular targeting. J. Control. Release.

[39] Dinache A., Tozar T., Smarandache A., Andrei I.R., Nistorescu S., Nastasa V., Staicu A., Pascu M.L., Romanitan M.O. (2020). Spectroscopic characterization of emulsions generated with a new laser-assisted device. Molecules.

[40] Jiang J.X., Török N.J. (2013). Liver injury and the activation of the hepatic myofibroblasts. Curr. Pathobiol. Rep..

[41] Tsuchida T., Friedman S.L. (2017). Mechanisms of hepatic stellate cell activation. Nat. Rev. Gastroenterol. Hepatol..

[42] Kang K.H., Qian Z.J., Ryu B., Karadeniz F., Kim D., Kim S.K. (2013). Hepatic fibrosis inhibitory effect of peptides isolated from navicula incerta on tgf-β1 induced activation of lx-2 human hepatic stellate cells. Prev. Nutr. Food Sci..

[43] Dewidar B., Meyer C., Dooley S., Meindl-Beinker A.N. (2019). Tgf-β in hepatic stellate cell activation and liver fibrogenesis-updated 2019. Cells.

[44] Chung J., Huda M.N., Shin Y., Han S., Akter S., Kang I., Ha J., Choe W., Choi T.G., Kim S.S. (2021). Correlation between oxidative stress and transforming growth factor-beta in cancers. Int. J. Mol. Sci..

[45] Foglia B., Cannito S., Bocca C., Parola M., Novo E. (2019). Erk pathway in activated, myofibroblast-like, hepatic stellate cells: A critical signaling crossroad sustaining liver fibrosis. Int. J. Mol. Sci..

[46] He X., Wang C., Wang H., Li L., Wang C. (2020). The function of mapk cascades in response to various stresses in horticultural plants. Front. Plant Sci..

[47] Son Y., Cheong Y.K., Kim N.H., Chung H.T., Kang D.G., Pae H.O. (2011). Mitogen-activated protein kinases and reactive oxygen species: How can ros activate mapk pathways?. J. Signal Transduct..

[48] Liebert M.A. (1982). Final report on the safety assessment of myristyl myristate and isopropyl myristate. Int. J. Toxicol..

[49] Jin S., Li S., Wang C., Liu J., Yang X., Wang P.C., Zhang X., Liang X.J. (2014). Biosafe nanoscale pharmaceutical adjuvant materials. J. Biomed. Nanotechnol..

[50] Vertuani S., Beghelli E., Scalambra E., Malisardi G., Copetti S., Dal Toso R., Baldisserotto A., Manfredini S. (2011). Activity and stability studies of verbascoside, a novel antioxidant, in dermo-cosmetic and pharmaceutical topical formulations. Molecules.

[51] Lopes L.B. (2014). Overcoming the cutaneous barrier with microemulsions. Pharmaceutics.

[52] Alberti I., Kalia Y.N., Naik A., Bonny J., Guy R.H. (2001). Effect of ethanol and isopropyl myristate on the availability of topical terbinafine in human stratum corneum, in vivo. Int. J. Pharm..

