# Supplementary material for: Enhanced In Vitro Efficacy of Verbascoside in Suppressing Hepatic Stellate Cell Activation via ROS Scavenging with Reverse Microemulsion
Source: Antioxidants (Basel). 2024 Jul 27;13(8):907. doi: 10.3390/antiox13080907 (PMC11351154; doi:10.3390/antiox13080907)
Supplement: Supplementary file 1 [file antioxidants-13-00907-s001.zip › antioxidants-3087618-supplementary.pdf]

Supplementary information

Supplementary Method 1. Isolation and purification of verbascoside

The air-dried whole plant samples of Pedicularis rex C. B. Clark (15.0 kg) were extracted with 20 L of 95% ethanol under reflux twice (each for 2h). Solvents in the combined extracts were then removed in vacuo. The ethanol extract obtained (530.2 g) was distributed in water and sequentially extracted with dichloromethane, ethyl acetate, and n-butanol to yield an n-butanol extract (120.4 g). The n-butanol extract was then applied to an HP20SS column eluted with MeOH–H_2O (0:1~1:0) to yield six fractions (FrB1~FrB6). Fr. B4 (60.5 g) was subjected to silica gel column chromatography eluted with CH_2Cl_2–MeOH (5:1) to obtain verbascoside (16.3 g), which was identified by its NMR data.

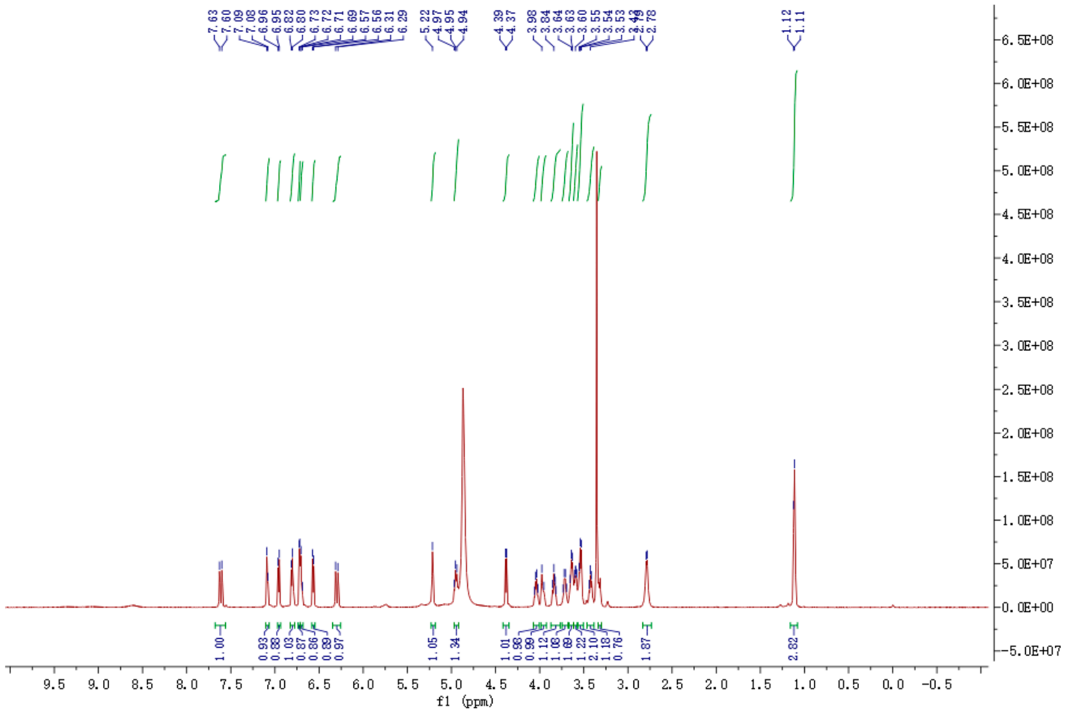

Supplementary Figure S1. ^{1}H-NMR of Verbascoside (CD3OD). (1): Amorphous powder. ^{1}H NMR (600 MHz, CD3OD) δ 7.62 (d, J = 15.8 Hz, 1H), 7.08 (d, J = 7.7 Hz, 1H), 6.96 (d, J = 8.2 Hz, 1H), 6.81 (d, J = 8.0 Hz, 1H), 6.72 (d, J = 4.1 Hz, 1H), 6.70 (d, J = 9.6 Hz, 1H), 6.57 (d, J = 7.9 Hz, 1H), 6.30 (d, J = 15.9 Hz, 1H), 5.21 (s, 1H), 4.95 (m, 1H), 4.38 (d, J = 7.8 Hz, 1H), 4.04 (dd, J = 15.9, 7.7 Hz, 1H), 3.83 (m, 1H), 3.64 (m, 1H) , 2.79 (d, J = 5.9 Hz, 2H), 1.12 (d, J = 5.9 Hz, 3H).

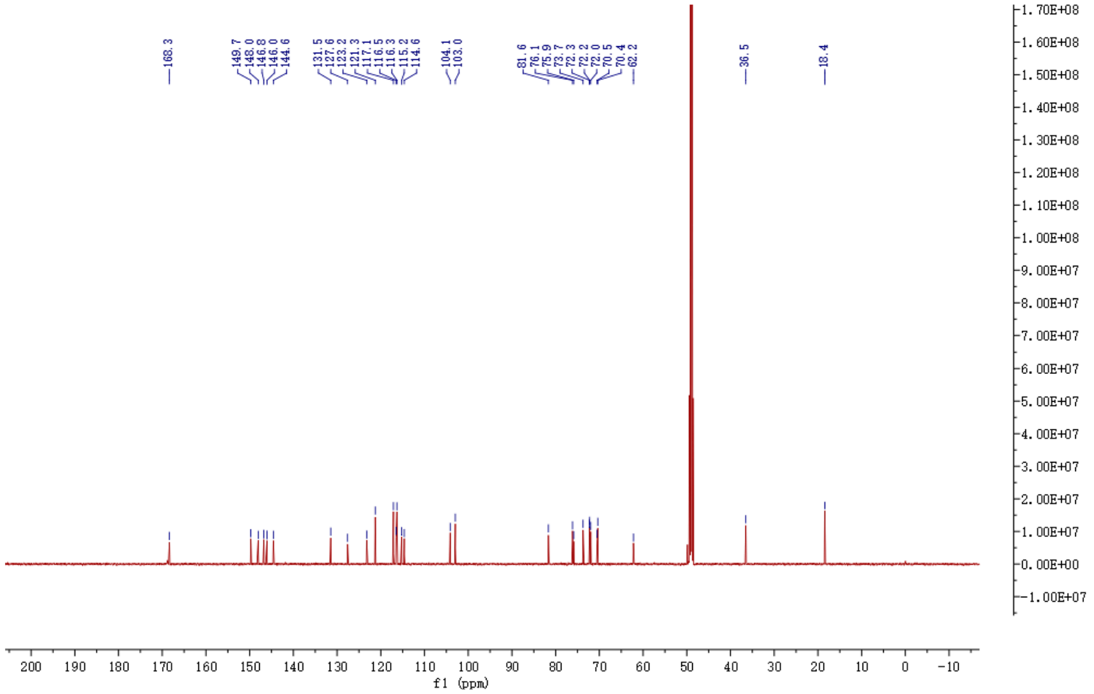

Supplementary Figure S2. ^{13}C-NMR of Verbascoside (CD3OD). (1): Amorphous powder. ^{13}C-NMR (151 MHz, CD3OD) δ 168.4, 149.7, 148.1, 146.8, 146.0, 144.6, 131.5, 127.6, 123.2, 121.3, 117.1, 116.5, 116.3, 115.2, 114.6, 104.1, 103.0, 81.7, 76.1, 75.9, 73.7, 72.3, 72.2, 72.0, 70.5, 70.4, 62.2, 36.5, 18.4.

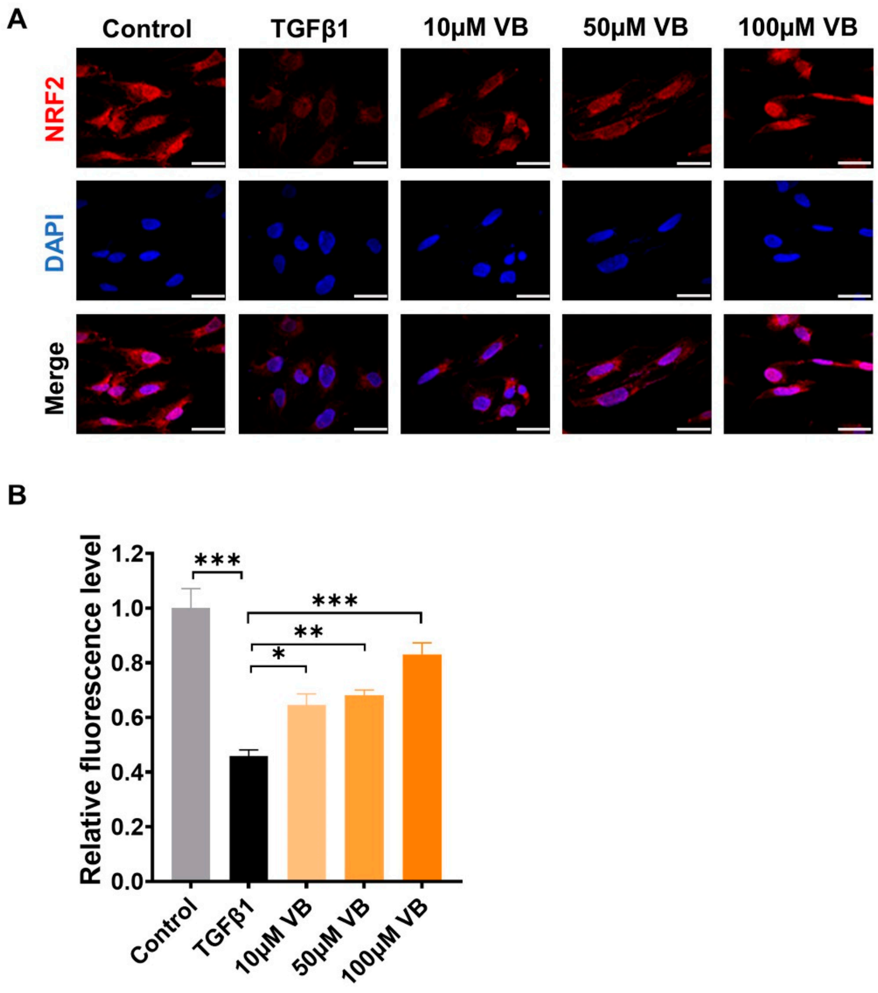

Supplementary Figure S3. The expression levels of Nrf2 protein in LX-2 cells were measured under different treatment conditions. LX-2 cells were pre-treated with VB at different concentrations for 6 hours, followed by treatment with TGFβ1 (5 ng/mL) for 24 hours. Inactive LX-2 cells served as the control group. Confocal images of LX-2 cells stained with Nrf2 antibody and DAPI. Scale bar, 20 μm. The data presented are representative of three independent replicate experiments. Data are presented as mean ± SEM (n=3), *: $p < 0.05$, **: $p < 0.01$, ***: $p < 0.001$.

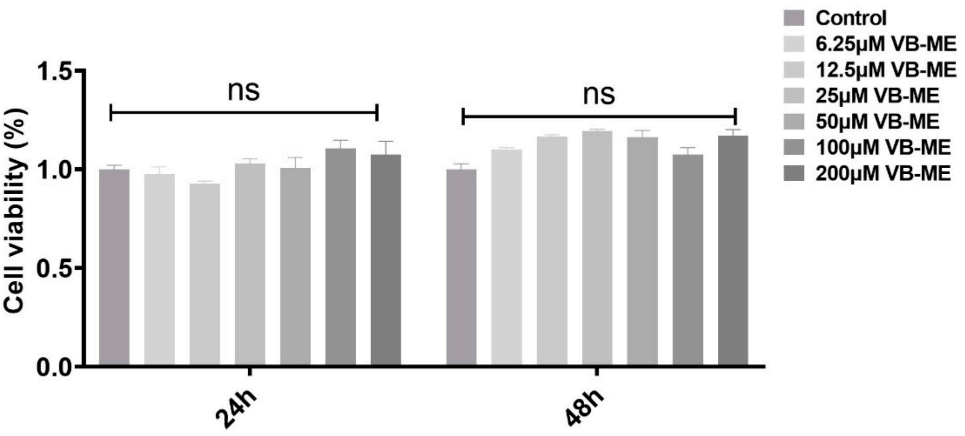

Supplementary Figure S4. LX-2 cells were treated with VB at concentrations of 3.125, 6.25, 12.5, 25, 50, and 100 μM for 24 hours and 48hours, and cell viability was assessed using the MTT assay. The control group was treated with PBS. The data presented are representative of three independent replicate experiments. Data are presented as mean ± SEM (n=4), with 'ns' indicating no significant differences.

Supplementary Table S1. Primers for RT-qPCR.

Gene	Forward sequence	Reverse sequence
COL1A1	ATCCCACCAATCACCTGCGT	TCATCGCACAACACCTTGCC
COL1A2	AAATATCGGCCCCGCTGGAA	GGCCTTTGGGTCCAGGGAAT
αSMA	ATGGTGCTCCCTGCATCTTC	CAGATCCAGACGCATGATGGCA
FN1	CTGGCCGAAAATACATTGTAAA	CCACAGTCGGGTCAGGAG
PDGFβ	CATGTGTCCTTGACCGGGGA	TGGGCACATAGTCCACCGAC
Nrf2	TCTTCTGTGCTGTCAAGGG	CTCTTTCCGTCGCTGACT
HPRT1	TGCTGAGGATTTGGAAAGGGTGTTT	GCACACAGAGGGCTACAATGTGATG
